# The Agreement Between Pulse Oximetry and Measured Arterial Oxygen Saturations in Postoperative Functionally Univentricular Patients

**DOI:** 10.3390/children13030415

**Published:** 2026-03-18

**Authors:** Fabio Savorgnan, Sebastian Acosta, Joshua Prabhu, Pranathi Pilla, Vikram Shah, Saul Flores, Rohit S. Loomba

**Affiliations:** 1 Department of Pediatrics, Division of Critical Care, Baylor College of Medicine, Houston, TX 77030, USA; fabio.savorgnan@bcm.edu (F.S.); saul.flores2@bcm.edu (S.F.); 2 Division of Emergency Medicine, Department of Pediatrics, Children’s Hospital of Michigan, Detroit, MI 48201, USA; 3 Baylor College of Medicine, Houston, TX 77030, USA; 4 Department of Computational Applied Mathematics and Operations Research, Rice University, Houston, TX 77005, USA; vs56@rice.edu; 5 Department of Pediatrics, Division of Cardiology, Northwestern University, Evanston, IL 60611, USA

**Keywords:** pediatrics, cardiology, pulse oximetry, arterial oxygen saturation, hypoxemia

## Abstract

**Highlights:**

**What are the main findings?**

- Pulse oximetry systematically overestimates arterial oxygen saturation in children with functionally univentricular circulation, with wide limits of agreement and worsening bias during hypoxemia.
- Arterial saturation level, rather than race, was the dominant determinant of measurement bias, with nonlinear overestimation increasing markedly at lower saturation ranges.

**What is the implication of the main finding?**

- In single-ventricle physiology, reliance on pulse oximetry alone may mask clinically significant hypoxemia, particularly when saturations fall below ~80%.
- Oxygen saturation values in congenital heart disease should be interpreted in a physiologic context, and confirmatory blood gas or adjunctive monitoring should be considered during clinical deterioration.

**Abstract:**

**Background**: Pulse oximetry is widely used to estimate arterial oxygen saturation, yet accuracy may vary for a number of reasons. Data on children with functionally univentricular circulation are limited. The primary aim of this study was to evaluate the agreement between arterial oxygen saturation measured by blood gas and pulse oximetry in children with functionally univentricular circulations. **Methods**: A retrospective analysis was performed of paired arterial blood gas and pulse oximetry oxygen saturation measurements following Norwood, Glenn, or Fontan procedures. Signed difference was defined as arterial oxygen saturation by blood gas—arterial oxygen saturation by pulse oximetry. Bland–Altman analyses, multivariable regressions, and generalized additive modeling were performed. **Results**: Mean bias was −4.9 percentage points, indicating pulse oximetry overestimated arterial saturation. The 95% limits of agreement were wide, from −20.7 to 10.8. The agreement was similar in Black and White patients. Fontan physiology demonstrated reduced overestimation by pulse oximetry by multivariable regression. Nonlinear modeling demonstrated more bias in agreement at lower arterial oxygen saturation levels, with arterial oxygen saturation levels explaining 50% of the variance. **Conclusions**: In functionally univentricular patients, pulse oximetry using the Nellcor MAXN-NS pulse oximeter (Medtronic, Dublin, Ireland) systematically overestimates arterial saturation, particularly in the setting of hypoxemia. Saturation level, rather than race, was the dominant determinant of bias.

## 1. Introduction

Pulse oximetry is a foundational component of pediatric and critical care monitoring, providing continuous noninvasive estimation of arterial oxygen saturation based on differential light absorption by oxygenated and deoxygenated hemoglobin [1,2]. The technology relies on the Beer-Lambert law to calculate the ratio of red and infrared light absorption, which is then translated into a saturation value via manufacturer-specific calibration curves derived from healthy volunteers [3]. Although widely adopted, pulse oximetry accuracy depends on device calibration, signal processing, and physiologic conditions at the time of measurement [4,5]. Pediatric studies have demonstrated modest systematic bias between pulse oximetry and arterial blood gas measurements, with widening limits of agreement at lower arterial oxygen saturation levels, particularly in children with congenital heart disease and critical illness [6,7]. Nonlinear proportional bias in hypoxemic ranges has been observed, suggesting reduced measurement stability when oxygen saturation declines below approximately eighty percent, a threshold where the empirical calibration curves of many commercial devices lose precision [8,9].

More recently, racial differences in pulse oximetry performance have received increased attention due to concerns that darker skin pigmentation may interfere with light transmission and lead to clinical overestimation of oxygenation [9]. Adult cohorts have demonstrated higher rates of occult hypoxemia among Black patients compared with White patients when pulse oximetry values appear clinically acceptable [8,9,10]. These findings have prompted a re-evaluation of pulse oximetry as a potential source of health disparities in critical care. Pediatric analyses have reported mixed findings, with variability observed across device platforms and saturation ranges [11]. Device-specific performance differences have been described, with some studies reporting variability between Nellcor and Masimo systems under certain conditions, suggesting that proprietary signal processing algorithms may handle pigment-related interference differently [12].

Children with functionally univentricular circulation represent a uniquely vulnerable population in whom accurate oxygen saturation assessment is central to clinical decision-making. Following the Norwood, Glenn, and Fontan procedures, these patients frequently reside in chronically hypoxemic states and exhibit complex circulatory physiology characterized by parallel circulation and altered systemic and pulmonary blood flow distribution [13]. In these patients, a delicate balance between systemic and pulmonary blood flow should be preserved as these work in parallel. Therefore, even a minor systematic bias can lead to inappropriate changes in vasoactive support or respiratory management [14]. Despite the utility of arterial oxygen saturation in guiding management, agreement between arterial blood gas saturation and pulse oximetry has not been comprehensively characterized across the distinct hemodynamic profiles of staged single-ventricle palliation. The primary aim of this study was to quantify agreement between arterial oxygen saturation and pulse oximetry in this unique cohort. A secondary aim of this study was to evaluate whether demographic characteristics, including race or physiologic stage, modulate measurement bias.

## 2. Methods

### 2.1. Study Design

This was a retrospective, single-center, observational study using paired arterial blood gas oxygen saturation and pulse oximetry oxygen saturation measurements obtained from patients with functionally univentricular circulation following the Norwood, Glenn, or Fontan procedure. The primary aim was to quantify the agreement between the two modalities. The secondary aim was to determine whether demographic or physiologic characteristics were associated with systematic bias or the magnitude of disagreement. The study was approved by the Baylor College of Medicine Institutional Review Board (protocol code H-52222 and date of approval 2 October 2025).

### 2.2. Variables of Interest

The primary variables of interest were arterial oxygen saturation obtained from arterial blood gas analysis and arterial oxygen saturation obtained by pulse oximetry using the Nellcor MAXN-NS pulse oximeter (Medtronic, Dublin, Ireland). Arterial oxygen saturation was obtained via point-of-care blood gas analysis in the pediatric cardiac intensive care unit. Paired measurements were defined by the close temporal proximity of the recorded arterial oxygen saturation at the time of the pulse oximeter recording and the arterial blood gas sampling. The precise window of the measurement was sixty seconds, during which the value was averaged over this time period. The stability requirements were not standardized in the extracted dataset. To ensure data representativeness, only arterial oxygen saturation values documented as stable signals by the bedside clinician were included.

Other variables for which data were collected were age (months), gender, circulatory physiology, race, Hispanic ethnicity, height, and weight. Gender was collected as male or female; circulatory physiology as Norwood, Glenn, or Fontan; race as White, Black, Asian, or other; Hispanic was collected as yes or no; height was captured in centimeters, and weight was captured in kg.

### 2.3. Patient Inclusion

All patients with functionally univentricular hearts who underwent Norwood, Glenn, or Fontan procedures were identified. Those with postoperative pairs of arterial blood gas oxygen saturation measurements and pulse oximetry arterial oxygen saturation were retained for inclusion. Those without a recorded race were excluded.

### 2.4. Statistical Analyses

All analyses were performed using paired arterial oxygen saturation and pulse oximetry saturation measurements. Continuous variables were summarized using median and interquartile range, while categorical variables were summarized using absolute frequency and percentages.

Agreement between arterial oxygen saturation and pulse oximetry arterial oxygen saturation measurements was evaluated using Bland–Altman methodology. For each paired observation, the signed difference was calculated as arterial oxygen saturation—pulse oximetry arterial oxygen saturation. The mean of the two measurements was calculated as the average of the arterial blood gas oxygen saturation measurement and the pulse oximetry arterial oxygen saturation. The mean signed difference was interpreted as the systematic bias. The standard deviation of the difference was calculated, and 95% limits of agreement were defined as the mean difference plus or minus 1.96 times the standard deviation. Bland–Altman analyses were utilized as they evaluate agreement by characterizing both central tendency and dispersion of measurement differences. From a practical perspective, this approach evaluates whether pulse oximetry consistently estimates the arterial oxygen saturation to be higher or lower than arterial blood gas analysis. Bland–Altman analyses were performed for the entire cohort and then stratified by race and circulatory physiology.

Next, a multivariable linear regression model was constructed using ordinary least squares estimation with heteroskedasticity-consistent robust standard errors. Two separate models were fit. The model used the signed difference as the dependent variable. Independent variables included age, gender, circulatory physiology, race, Hispanic ethnicity, height, and weight. Categorical variables were modeled using treatment coding with the most prevalent category serving as the reference group. Model fit was quantified using the coefficient of determination. This was done to help estimate the independent association between each covariate and measurement disagreement while holding all other variables constant.

To evaluate nonlinear proportional bias across the arterial oxygen saturation spectrum, a generalized additive modeling framework was employed. Cubic spline basis functions were generated for mean saturation, with six degrees of freedom included in the model as smooth terms. Interaction terms were constructed between each spline basis function and age, gender, circulatory physiology, race, Hispanic ethnicity, height, and weight. The final model, therefore, allowed the relationship between mean saturation and signed difference to vary flexibly across subgroups. The model was estimated using ordinary least squares, and model fit was assessed using the coefficient of determination. Predicted bias values were generated from the generalized additive model across a range of mean saturation values spanning the observed data. Model-implied 95% limits of agreement at selected saturation levels were calculated by combining predicted mean bias with the model residual standard deviation. These estimates provide an interpretable representation of how measurement disagreement changes across clinically relevant oxygen saturation ranges.

The generalized additive model relaxes the assumption of linearity and permits complex nonlinear relationships between saturation level and measurement bias. The inclusion of interaction terms allows the shape of this nonlinear curve to differ by demographic or physiologic characteristics. In simpler terms, this approach evaluates whether arterial oxygen saturation by pulse oximetry behaves differently at lower versus higher arterial oxygen saturation levels and whether those differences vary by race or circulatory physiology.

All statistical analyses were done using the statsmodels V0.13.2 library in the Python environment v3.8.3. A *p*-value of less than 0.05 was considered statistically significant. Any use of the word “significant” or “significantly” in this manuscript will refer to statistical significance unless explicitly stated as clinical significance.

## 3. Results

### 3.1. Cohort Characteristics

A total of 5263 paired measurements were analyzed. These were gathered from 368 unique patients. At a patient level, the median age was 9.8 months (interquartile range 2.1 to 55.8), the median weight was 7.9 kg (interquartile range 4.3 to 16.0 kg), and the median height was 67.6 cm. Of the 368 patients, 308 (84%) were White, 38 (10%) were Black, 19 (5%) were Asian, and 3 (1%) were another race. Of the 368 patients, 159 (43%) had Fontan physiology, 114 (31%) had Glenn physiology, and 95 (26%) had Norwood physiology (Table 1).

### 3.2. Bland–Altman Analyses

**Figure 1:**
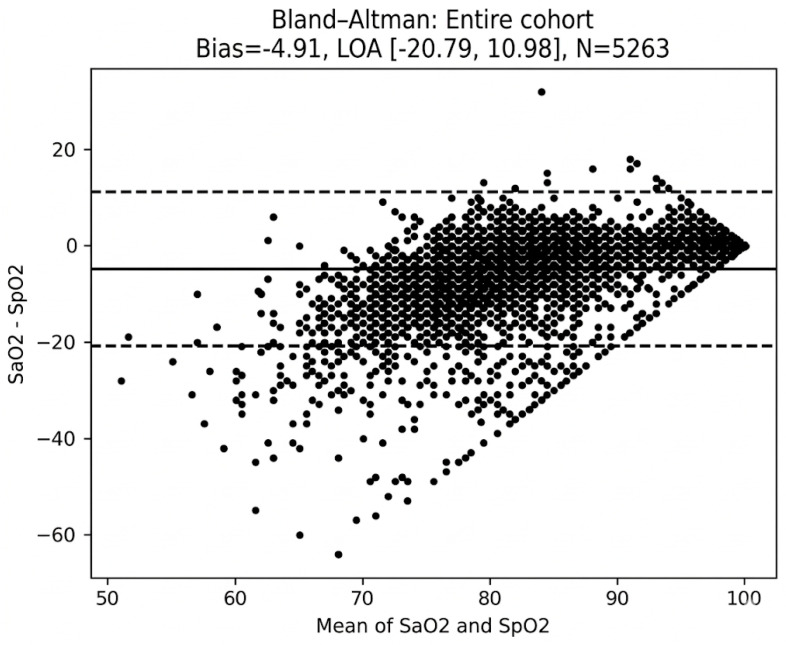
Bland–Altman Plot of Cohort.

Across the entire cohort, mean bias was negative 4.9 percentage points, indicating that pulse oximetry overestimated arterial saturation by that amount. The 95% limits of agreement were −20.7 to 10.9 (Figure 1).

Race-stratified resulted demonstrated bias of −5.0 percentage points in White patients (95% limits of agreement −20.7 to 10.7), −5.2 percentage points in Black patients (95% limits of agreement −22.6 to 12.2), −1.7 percentage points in Asian patients (95% limits of agreement −16.0 to 12.7), and −1.3 percentage points in patients with other race (95% limits of agreement (−8.0 to 5.4). Thus, pulse oximetry overestimated arterial saturation in all races.

Circulatory physiology-specific bias was −5.8 percentage points in Norwood patients (95% limits of agreement −19.6 to 7.9), −5.7 percentage points in Glenn patients (95% limits of agreement −20.8 to 9.4), and −2.7 percentage points in Fontan patients (95% limits of agreement −21.1 to 15.5). Thus, pulse oximetry overestimated arterial saturation in all circulatory physiologies (Table 2).

While the mean bias provides a population-level view of device performance, the wide 95% limits of agreement highlight the significant interval of uncertainty for individual clinical encounters. A limit of agreement extending to −20.8% in Glenn patients implies that for a significant portion of measurements, the pulse oximeter may provide a value that is dangerously disconnected from the patient’s true physiological state.

### 3.3. Regression Analyses

For the signed difference, the coefficient of determination was 0.04. Age in months demonstrated a coefficient of 0.008 percentage points per month (*p* = 0.18), height demonstrated a coefficient of −0.004 per centimeter (*p* = 021), and weight demonstrated a coefficient of −0.031 (*p* = 0.14). Relative to the Norwood circulatory physiology, Glenn physiology did not significantly impact the signed difference (*p* = 0.65) while Fontan physiology significantly reduced overestimation of arterial oxygen saturation by pulse oximetry (*p* < 0.01). Relative to the White race, the Black race demonstrated no significant difference in signed difference (*p* = 0.29), while the Asian race was associated with less overestimation of arterial oxygen saturation by pulse oximetry (*p* < 0.01).

### 3.4. Generalized Additive Model Analyses

The generalized additive model incorporating spline terms and interaction terms demonstrated a coefficient of determination of 0.50, indicating the model explained 50% of the variance in the difference between arterial blood gas oxygen saturation and arterial oxygen saturation by pulse oximetry.

**Figure 2:**
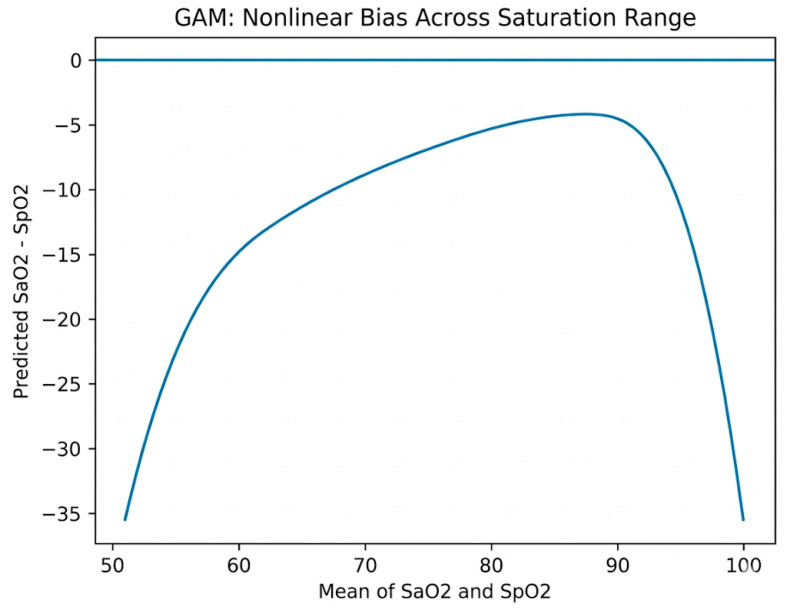
Generalized Additive Model of Cohort.

Predicted bias varied substantially across the saturation spectrum. At a mean arterial oxygen saturation of 55%, predicted bias was approximately −30 percentage points. At a mean arterial oxygen saturation of 65%, predicted bias was approximately −15 percentage points. At a mean arterial oxygen saturation of 75%, predicted bias was approximately −8 percentage points. At a mean arterial oxygen saturation of 85%, predicted bias was approximately −4 percentage points. At 90%, predicted bias approached −2 percentage points (Figure 2).

Race-specific interaction curves were largely overlapping across mean saturations, indicating no significant difference in predicted bias by race. The arterial oxygen saturation by blood gas explained substantially more variance in bias than other variables.

## 4. Discussion

Pulse oximetry systematically overestimated arterial oxygen saturation in this cohort of 5263 paired measurements obtained from 368 unique patients with functionally univentricular circulation. On average, the arterial oxygen saturation by pulse oximetry was 4.9 percentage points higher than that measured by arterial blood gas analysis, with wide 95% levels of agreement (−20.7 to 10.9). The systematic overestimation of oxygenation by pulse oximetry is particularly concerning in the management of the parallel circulation. In these patients, the balance between systemic and pulmonary blood flow is delicate, and a false reading can lead to a delay in recognizing a low-output state, which can exacerbate conditions like lactic acidosis or mesenteric ischemia.

Regression analyses demonstrated that Fontan physiology and Asian race were associated with less overestimation of arterial oxygen saturation by pulse oximetry. The regression model only explained 3.9% of the variance. The generalized additive model explained 50% of the variance and demonstrated that the arterial oxygen saturation level itself was the dominant determinant of measurement disagreement, with pulse oximetry overestimating the arterial saturation by a greater degree at lower arterial oxygen saturations.

Bias was least pronounced in the Fontan cohort. Studies have shown that both Norwood and Fontan procedures have had similar survival rates in patients [15]. The improved agreement in Fontan patients may be attributed to higher baseline saturations and more predictable pulsatile flow, which likely enhances the signal processing capabilities of the pulse oximeter compared to the more volatile neonatal period.

Previous pediatric studies evaluating agreement between arterial oxygen saturation and arterial oxygen saturation by pulse oximetry have also demonstrated modest mean bias, with greater mean bias at lower arterial oxygen saturation levels. In infants and children with congenital heart disease, the mean difference between arterial blood gas oxygen saturation and pulse oximetry arterial oxygen saturation has ranged from −2 to −6, with wide limits of agreement in hypoxemic states, thus showing similar overestimation of arterial saturation by pulse oximetry as the current study [5,16]. Several investigations have highlighted nonlinear proportional bias below arterial oxygen saturations of approximately 80% [5,6]. The findings from the current study are consistent with these previously published findings, with predicted bias from the generalized additive model approaching −15 percentage points by an arterial oxygen saturation of 65%.

The progressive widening of measurement disagreement at lower saturation levels likely reflects a combination of physiologic and optical factors. Although the steep portion of the oxyhemoglobin dissociation curve may amplify physiologic variability in this range, the accuracy of pulse oximetry in the setting of hypoxemia is also likely influenced by altered light absorption, reduced signal-to-noise ratio, and device calibration limitations at low saturation levels [2,3]. Commercial pulse oximeters are primarily calibrated using healthy volunteers and have limited empirical calibration in arterial oxygen saturation ranges below approximately 70% [1,2,5]. As arterial oxygen saturation decreases, the ratio of deoxygenated to oxygenated hemoglobin shifts, optical absorption characteristics change, and the pulsatile arterial signal may become proportionally smaller relative to the background signal. Venous pulsation, motion artifact, and low peripheral perfusion may contribute additional distortion [2]. In functionally univentricular circulation, where systemic venous pressures may be elevated and perfusion may be variable, these optical and hemodynamic factors may further degrade measurement stability. Accordingly, the observed nonlinear bias should not be attributed solely to the oxyhemoglobin dissociation curve but rather to the combined effects of physiologic nonlinearity and optical measurement limitations [3,5].

Recent literature has emphasized racial disparities in pulse oximetry performance, particularly the risk of occult hypoxemia among Black patients. Adult studies have demonstrated increased rates of unrecognized hypoxemia in Black patients compared with White patients when pulse oximetry values appear within acceptable ranges [8,9,10,11]. Pediatric analyses have reported similar findings, although they have been less consistent across device platforms [11]. Comparative device analyses have suggested variability between Nellcor and Masimo systems with regard to race-specific accuracy [11]. Mechanistically, differences in light absorption related to skin pigmentation have been proposed as contributors to differential accuracy.

In contrast to adult cohorts with predominantly normal arterial oxygen saturation levels, the current study did not demonstrate a clinically meaningful independent association between Black race and systematic bias after adjustment. Race-stratified Bland–Altman analyses demonstrated nearly identical mean bias between Black and White patients, at approximately −5 percentage points in both groups. Nonlinear interaction modeling demonstrated largely overlapping bias curves, suggesting that in functionally univentricular circulatory physiologies, arterial oxygen saturation levels exert a substantially greater influence on pulse oximetry bias than race. In this hypoxemic pediatric population, the magnitude of arterial desaturation and associated optical limitations appear to be more influential than race-associated variability.

The functionally univentricular population represents a unique physiologic context. These patients are chronically hypoxemic until Fontan palliation. Functionally univentricular multi-distributive circulation and functionally univentricular Glenn circulation exhibit altered systemic and pulmonary blood flow distribution, and variable systemic blood flow. Prior pediatric validation studies have not extensively examined pulse oximetry performance across staged functionally univentricular palliation [5]. The current study demonstrates that predicted bias increases sharply as arterial oxygen saturation declines, reinforcing that nonlinear physiologic and optical effects dominate measurement performance in cyanotic congenital heart disease.

These findings carry important clinical implications. Within an oximetric framework, systemic oxygen delivery is what maintains organ function and modulates morbidity and mortality. Median arterial oxygen saturation in this cohort was 82%, with rapidly decreasing accuracy of pulse oximetry below this saturation. Such discrepancies can materially alter the interpretation of oxygen delivery and oxygen extraction. In patients with clinical deterioration, reliance solely on pulse oximetry may mask clinically significant hypoxemia. Other ancillary monitoring, such as venous oxygen saturation monitoring or near-infrared spectroscopy, can be particularly helpful in these settings. Additionally, when clinical uncertainty exists, particularly in the setting of hypoxemia, arterial blood gas analysis provides a much more accurate assessment of arterial oxygen saturation when necessary.

Strengths of this study include the large number of paired measurements, inclusion of 368 unique patients across various stages of functionally univentricular palliation, and integration of traditional agreement analysis with flexible nonlinear modeling. The use of generalized additive modeling allowed for quantification of saturation-dependent bias across the full observed range. Stratified analyses permitted direct comparison by race and circulatory stage.

Limitations of this study include its retrospective design, which may limit generalizability. Additionally, due to the retrospective nature of the data extraction, we were unable to objectively assess waveform quality or verify the exclusion of motion artifacts for every paired measurement. While clinical protocol dictates that the arterial oxygen saturation should only be documented during stable signal acquisition, unmeasured signal interference may have contributed to the wide limits of agreement observed, particularly in the more hemodynamically complex Norwood and Glenn cohorts. This is particularly important to note as different centers utilize different makes and models of pulse oximetry devices. Because this study focused exclusively on the Nellcor MAXN-NS system, these findings may not be generalizable to other pulse oximetry platforms that utilize different signal processing algorithms or calibration curves. Also, while a single device type was used, we did not record probe-specific changes or weight-based selection criteria, which may introduce further unmeasured variability. Second, we did not account for anatomic probe location or perfusion status; the absence of data on lactate, temperature, or vasoactive support limits our ability to determine how hemodynamic instability contributed to measurement disparity. Third, physiological confounders such as pH and temperature were not included in our models, which may alter the oxyhemoglobin dissociation curve and the resulting arterial oxygen saturation relationship. This dataset lacked information on respiratory support, meaning we could not determine the impact of mechanical ventilation on measurement stability.

Repeated measurements per patient may introduce within-subject correlation that was not addressed using hierarchical techniques. Race was treated as recorded in the medical record and reflects a social construct rather than a biological variable. Additionally, this was used as a surrogate for skin tone, which could not be directly assessed for this study. Furthermore, the interpretation of our findings regarding race must be viewed within the context of our cohort’s composition. The study population was predominantly White (84%), with Black patients representing only 10% of the unique patients identified. This limited representation of Black and other minority groups may reduce the statistical power to detect more subtle race-based differences in pulse oximetry accuracy within this specific clinical population. Consequently, our conclusion that saturation level—rather than race—is the primary driver of bias should be validated in more diverse cohorts of children with functionally univentricular circulation. Additionally, this study only includes patients with functionally univentricular hearts, and these findings cannot be generalized to biventricular patients. All findings in this study are associations and not causal. This study and its findings are exploratory and should be treated as hypothesis-generating.

## 5. Conclusions

In functionally univentricular patients, pulse oximetry using the Nellcor MAXN-NS pulse oximeter (Medtronic, Dublin, Ireland) systematically overestimates arterial saturation, particularly in the setting of hypoxemia. Saturation level, rather than race, was the dominant determinant of bias.

## Figures and Tables

**Table 1:** Race and Procedure Breakdown of Cohort.

**Race**				
	White	Black	Asian	Another Race
	308 (83.7%)	38 (10.3%)	19 (5.2%)	3 (0.08%)
**Procedure**				
	Fontan	Glenn	Norwood	
	159 (43.2%)	114 (31.0%)	95 (25.8%)	

**Table 2:** Bias and Agreement Between Arterial Oxygen Saturation by Race and Surgical Procedure.

Race	Mean Bias (%)	95% Limits of Agreement (%)
White	−5.0	−20.7 to 10.7
Black	−5.2	−22.6 to 12.2
Asian	−1.7	−16.0 to 12.7
Other Race	−1.3	−8.0 to 5.4
**Surgical Procedure**		
Norwood	−5.8	−19.6 to 7.9
Glenn	−5.7	−20.8 to 9.4
Fontan	−2.7	−21.1 to 15.5

## Data Availability

The original contributions presented in this study are included in the article. Further inquiries can be directed to the corresponding author.
